# Supplementary material for: Effectiveness of an inactivated Covid-19 vaccine with homologous and heterologous boosters against Omicron in Brazil
Source: Nat Commun. 2022 Oct 6;13:5536. doi: 10.1038/s41467-022-33169-0 (PMC9537178; doi:10.1038/s41467-022-33169-0)
Supplement: Supplementary file 1 — Supplementary Information [file 41467_2022_33169_MOESM1_ESM.pdf]

Supplementary material

Supplementary Table 1. Data sources

Supplementary Table 2. General characteristics of who received homologous or heterologous booster

Supplementary Figure 1. Number of SARS-CoV-2 RT-PCR and rapid antigen tests in symptomatic individuals performed in Brazil since January 2021.

Supplementary Figure 2. Study flow chart showing inclusion of cases and controls for the primary analysis

Supplementary Table 3. Unadjusted vaccine effectiveness of CoronaVac and homologous or heterologous booster against symptomatic Covid-19 and hospital admissions or deaths in adults in Brazil

Supplementary Table 4. Adjusted vaccine effectiveness of CoronaVac and homologous or heterologous booster against symptomatic Covid-19 and hospital admissions or deaths in adults in Brazil

Supplementary Table 5. Unadjusted vaccine effectiveness of homologous or heterologous booster against symptomatic Covid-19 and Covid-19 hospital admissions or deaths in adults stratified by age during Omicron period in Brazil

Supplementary Table 6. Characteristics of adults in Brazil, who were selected into case test negative pairs for the CoronaVac analysis during the Delta period (September 6, 2021 to December 14, 2021) and Omicron period (December 25, 2021 to April 22, 2022), for the analysis of relative vaccine effectiveness

Supplementary Table 7. Unadjusted vaccine effectiveness of a homologous and heterologous booster dose, relative to primary vaccination with CoronaVac during the period greater or equal to 180 days after the 2nd dose during Omicron period

Supplementary Table 8. Characteristics of adults in Brazil, who were selected into case test negative pairs for the analysis of vaccine effectiveness during the Delta period (September 6, 2021 to December 14, 2021) and the Omicron period (December 25, 2021 to April 22, 2022) - Sensitivity analysis for matching

Supplementary Table 9. Sensitivity analysis of matching strategy to evaluate the adjusted vaccine effectiveness of CoronaVac and homologous or heterologous booster against symptomatic Covid-19 using RT-PCR or Antigen tests

Supplementary Table 10. Sensitivity analysis of matching strategy to evaluate the adjusted vaccine effectiveness of CoronaVac and homologous or heterologous booster against Severe Covid-19 using RT-PCR or Antigen tests

Supplementary Table 11. Adjusted vaccine effectiveness of CoronaVac and homologous or heterologous booster against severe Covid-19 in adults in Brazil: main analysis vs sensitivity analysis of the severe COVID-19 definition

Supplementary Table 12. Adjusted vaccine effectiveness of homologous or heterologous booster against severe Covid-19 in adults stratified by age during Omicron period in Brazil: main analysis vs sensitivity analysis of the severe COVID-19 definition

Supplementary Table 13. Adjusted vaccine effectiveness of a homologous and heterologous booster dose, relative to primary vaccination with CoronaVac during the period greater or equal to 180 days after the 2nd dose during Omicron period, further adjusted by month of second dose (sensitivity analysis)

Supplementary Figure 3. Flow chart showing inclusion of cases and controls for the sensitivity analysis including only RT-PCR SARS-CoV-2 tests

Supplementary Table 14. Characteristics of adults in Brazil, who were selected into case-test negative pairs for the sensitivity analysis including only RT-PCR tests during the Delta period

(September 6, 2021 to December 14, 2021) and Omicron period (December 25, 2021 to April 2, 2022)

Supplementary Table 15. Adjusted vaccine effectiveness of CoronaVac and homologous or heterologous booster against symptomatic Covid-19 and Covid-19 hospital admission or deaths in adults in Brazil, from the sensitivity analysis including RT-PCR SARS-CoV-2 tests only

Supplementary Table 16. Characteristics of adults in Brazil, who were selected into case-test negative pairs for the sensitivity analysis using RT-PCR SARS-CoV-2 tests only, during the Delta period (September 6, 2021 to December 14, 2021) and Omicron period (December 25, 2021 to April 22, 2022), for the analysis of relative vaccine effectiveness

Supplementary Table 17. Adjusted vaccine effectiveness of homologous or heterologous booster against symptomatic Covid-19 and Covid-19 hospital admission or deaths in adults stratified by age during Omicron period in Brazil from the sensitivity analysis including RT-PCR SARS-CoV-2 tests only

Supplementary Table 18. Adjusted vaccine effectiveness of homologous and heterologous booster relative to those at least 180 days after the second dose of a primary series of CoronaVac during the Omicron period, from the sensitivity analysis including RT-PCR SARS-CoV-2 tests only

Supplementary Table 1. Data sources

Data	Source	Extracted	Censored	Content
Suspected mild COVID-19	e-SUS notifica	29/04/2022	22/04/2022	COVID-19 suspected individuals of any age in Brazil, from public and private sectors, with mild clinical presentation, including those asymptomatic, not tested, those tested negative and tested positive. COVID-19 suspected cases notification is compulsory.
Suspected Severe COVID-19	SIVEP-Gripe	29/04/2022	22/04/2022	Severe Acute Respiratory Infections (SARI) at any age in Brazil, from public and private sectors. Includes those not tested, those tested negative and those tested positive. It also includes SARI caused by other respiratory viruses, other aetiologies and those without an etiology. During COVID-19 pandemic, the official system to which COVID-19 hospital admissions and deaths must be notified. COVID-19 suspected cases notification is compulsory.
COVID-19 vaccination status	SI-PNI	29/04/2022	22/04/2022	National database from the Brazilian National Programme of Vaccination. COVID-19 vaccines notification is compulsory.
Variants of Concern in Brazil	GISAID	09/05/2022	22/04/2022	Public genomic repository data

Supplementary Table 2. General characteristics of who received homologous or heterologous booster

	Homologous booster (CoronaVac) (n=69,111)	Heterologous booster (BNT152b2) (n=984,444)	SMD
**Demographics**			
**Age, mean (SD), years**	65.1 (21.5)	53.2 (19.1)	0.602
**Age categories, n (%)**			0.645
**18-39 years**	12890 (18.7)	321694 (32.7)	
**40-59 years**	8313 (12.0)	227987 (23.2)	
**60-79 years**	31925 (46.2)	380315 (38.6)	
**≥80 years**	15983 (23.1)	54448 (5.5)	
**Male sex, n (%)**	27431 (39.7)	336163 (34.1)	0.115
**Self-reported race†, n (%),**			0.399
**White/Branca**	42677 (61.8)	461276 (46.9)	
**Mixed/Pardo**	9464 (13.7)	278390 (28.3)	
**Black/Preta**	1750 (2.5)	34897 (3.5)	
**Asian/ Amarela**	652 (0.9)	15873 (1.6)	
**Indigenous/Indigena**	25 (0.0)	2377 (0.2)	
**Missing**	14543 (21.0)	191631 (19.5)	
**Region of residence**			1.234
**North**	278 (0.4)	49932 (5.1)	
**Northeast**	302 (0.4)	173982 (17.7)	
**Central-West**	350 (0.5)	75923 (7.7)	
**Southeast**	66494 (96.2)	489057 (49.7)	
**South**	1687 (2.4)	195550 (19.9)	
**Reported number of chronic comorbidities‡, n (%)**			0.221
**None**	56094 (81.2)	875843 (89.0)	
**One or two**	12457 (18.0)	104856 (10.7)	
**Three or more**	560 (0.8)	3745 (0.4)	
**Prior SARS-CoV-2 exposure**			
**Previous symptomatic events notified to the surveillance system¶, n (%)**	14381 (20.8)	253801 (25.8)	0.118

**Positive SARS-CoV-2 test result††, n (%)**	3268 (4.7)	52378 (5.3)	0.027
**Interval between symptoms onset and RT-PCR/Antigen testing, median (p25-p75), days**	3 [2, 4]	3.00 [2, 4]	0.038
**Interval between third dose and testing, median (p25-p75), days**	93 [50, 116]	87 [56, 105]	0.123
**Omicron period**	67747 (98.0)	966813 (98.2)	0.013

RT-PCR=reverse transcription polymerase chain reaction; SMD=standardized mean difference; SD=standard deviation; SMD=standardized mean difference. † Race/skin colour as defined by the Brazilian national census bureau (Instituto Nacional de Geografia e Estatísticas). ‡ Comorbidities included cardiovascular, or renal conditions, diabetes, chronic respiratory disorder, obesity, or immunosuppression. ¶ Reported illness with covid-19 associated symptoms in eSUS and SIVEP-Gripe databases before the start of study on 06 September 2021.. †† Defined as a positive SARS-CoV-2 RT-PCR or antigen detection test result before the start of study on 06 September 2021.

Supplementary Figure 1. Number of SARS-CoV-2 RT-PCR and rapid antigen tests in symptomatic individuals performed in Brazil since January 2021.

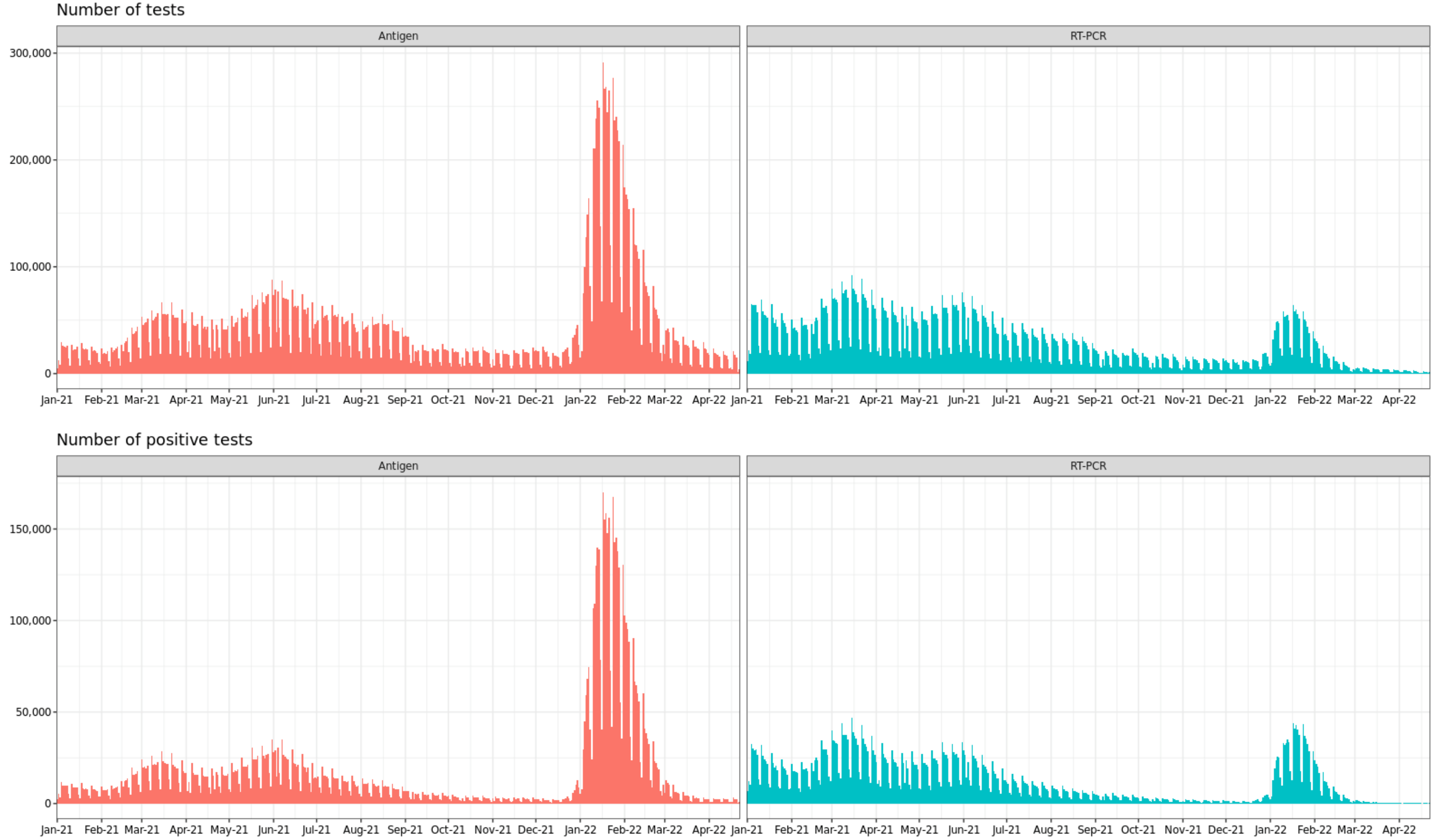

Red refers to rapid antigen test for SARS-CoV-2; green to RT-PCR test for SARS-CoV-2

Supplementary Figure 2. Study flow chart showing inclusion of cases and controls for the primary analysis

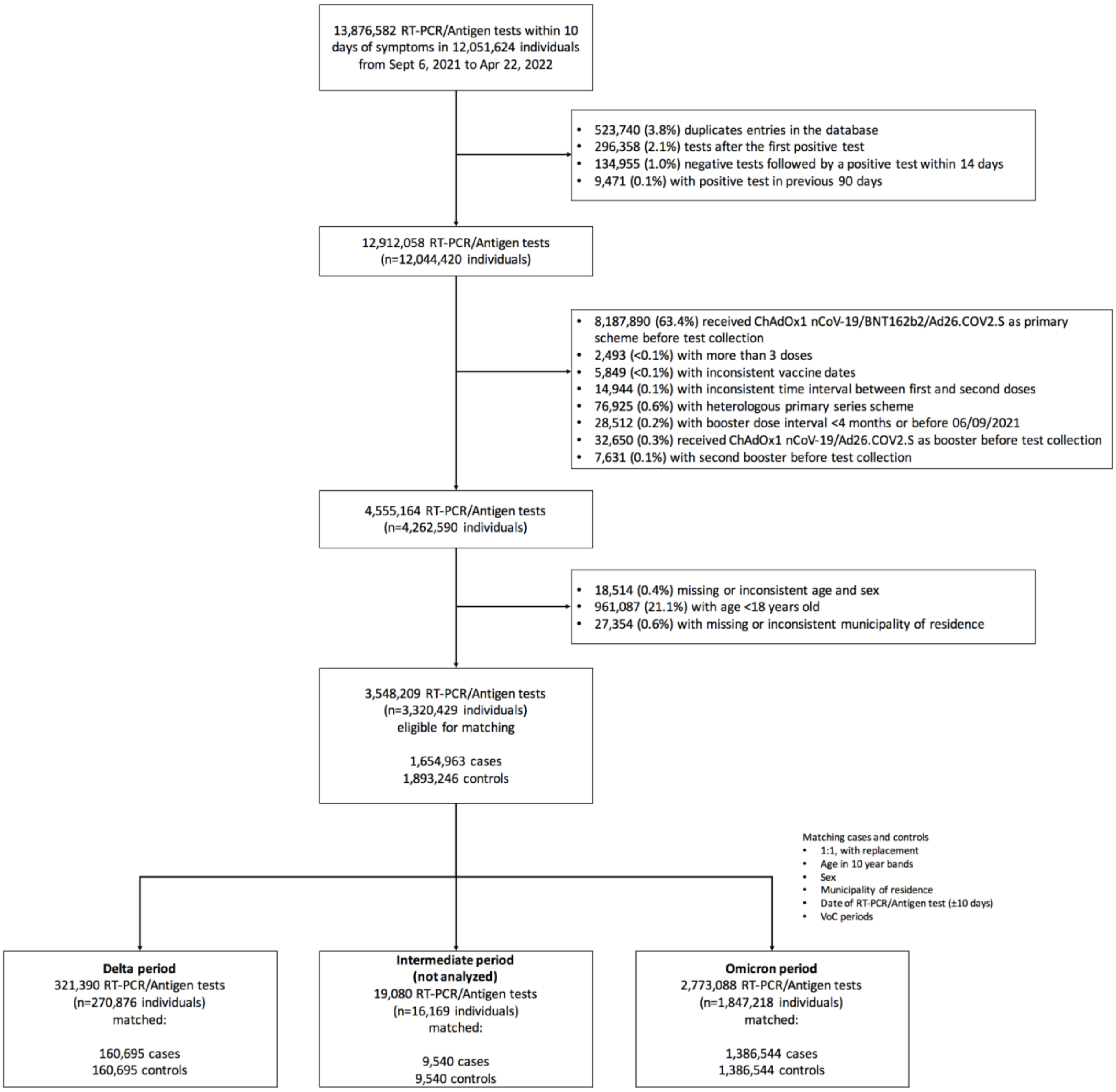

Supplementary Table 3. Unadjusted vaccine effectiveness of CoronaVac and homologous or heterologous booster against symptomatic Covid-19 and hospital admissions or deaths in adults in Brazil

	**Symptomatic COVID-19**		**Hospitalization or death**	
	Delta	Omicron	Delta	Omicron
**Not vaccinated**	Reference	Reference	Reference	Reference
**Single dose, within 0-13 days**	5% (-1-10.7)	25.8% (20.4-30.9)	-1% (-28.7-27.3)	0.8% (-50.9-51.7)
**Single dose, ≥14 days**	29% (26.9-31.1)	18.4% (17.3-19.4)	44% (36.8-50.4)	42.4% (36-48.1)
**Two doses, within 0-13 days**	40.1% (37.1-43)	23% (19-26.8)	79% (71.4-84.6)	22.5% (-38.4-63)
**Two doses, 14-59 days**	52.4% (51-53.7)	29.7% (28.2-31.2)	86.6% (83.8-88.9)	56.2% (42.7-66.5)
**Two doses, 60-179 days**	38.7% (37.2-40.1)	5.4% (4.6-6.2)	70.4% (68.1-72.5)	63.6% (60.2-66.7)
**Two doses, ≥180 days**	34.2% (32.5-35.9)	4.5% (3.5-5.5)	55.6% (52.1-59)	53.1% (50-55.9)
**Third dose of CoronaVac, 0-7 days**	40.1% (20.3-54.9)	10.2% (2.4-17.4)	73.4% (56.9-83.6)	77.6% (42.7-91.2)
**Third dose of CoronaVac, 8-59 days**	54.2% (47.2-60.3)	7.4% (4.3-10.3)	69% (60.1-75.9)	77.4% (70.7-82.5)
**Third dose of CoronaVac, ≥60 days**	50.7% (27.2-66.7)	-13.2% (-15.2--11.1)	71.2% (47.2-84.3)	55.8% (51.9-59.4)
**Third dose of BNT162b2, 0-7 days**	43% (38.9-46.8)	16.7% (14.8-18.5)	76.5% (71.6-80.5)	63.5% (53.6-71.3)
**Third dose of BNT162b2, 8-59 days**	86.8% (86.1-87.5)	56.4% (55.9-56.9)	90.8% (89.4-91.9)	86.2% (85-87.4)
**Third dose of BNT162b2, ≥60 days**	82.8% (79.4-85.7)	31.1% (30.5-31.8)	88.2% (81.6-92.4)	85.5% (84.6-86.4)

Supplementary Table 4. Adjusted vaccine effectiveness of CoronaVac and homologous or heterologous booster against symptomatic Covid-19 and hospital admissions or deaths in adults in Brazil

	Symptomatic COVID-19		Hospitalization or death	
	Delta	Omicron	Delta	Omicron
**Not vaccinated**	Reference	Reference	Reference	Reference
**Single dose, within 0-13 days**	2.2% (-4-8.1)	24.6% (19.1-29.8)	1.7% (-28.4-30.8)	-3.1% (-57.5-54.7)
**Single dose, ≥14 days**	27.2% (25.1-29.3)	17% (15.9-18)	46.6% (38.9-53.3)	47.5% (40.7-53.5)
**Two doses, within 0-13 days**	38.5% (35.3-41.5)	21.3% (17.3-25.2)	76.7% (67.3-83.4)	14.8% (-46-60.8)
**Two doses, 14-59 days**	51.3% (49.9-52.7)	28.1% (26.5-29.6)	86.5% (83.4-88.9)	56.1% (40.6-67.5)
**Two doses, 60-179 days**	37.6% (36.1-39.1)	3.9% (3.1-4.8)	71% (68.5-73.2)	61.4% (57.4-65)
**Two doses, ≥180 days**	34% (32.3-35.7)	6.3% (5.3-7.3)	60.9% (57.3-64.2)	57.6% (54.4-60.6)
**Third dose of CoronaVac, 0-7 days**	44.7% (26.2-58.5)	9.6% (1.7-16.9)	80.8% (67.7-88.6)	75.6% (26.7-91.8)
**Third dose of CoronaVac, 8-59 days**	57.1% (50.4-62.9)	8.6% (5.6-11.5)	75.9% (67.8-81.9)	73.6% (63.9-80.7)
**Third dose of CoronaVac, ≥60 days**	53.5% (30.9-68.6)	-2.9% (-5.2--0.6)	75.6% (52.8-87.4)	67.8% (64.3-71)
**Third dose of BNT162b2, 0-7 days**	42.6% (38.5-46.5)	16.6% (14.7-18.4)	80.4% (75.8-84)	62.6% (50.7-71.6)
**Third dose of BNT162b2, 8-59 days**	86.7% (86-87.4)	56.8% (56.3-57.3)	92.3% (91-93.4)	86% (84.5-87.4)
**Third dose of BNT162b2, ≥60 days**	83.2% (79.8-86.1)	33.8% (33.2-34.4)	90.8% (84.8-94.4)	86.4% (85.4-87.3)

Supplementary Table 5. Unadjusted vaccine effectiveness of homologous or heterologous booster against symptomatic Covid-19 and Covid-19 hospital admissions or deaths in adults stratified by age during Omicron period in Brazil

	<60 years		60-74 years		≥75 years	
Symptomatic	Controls/Cases	VE (95% CI)	Controls/Cases	VE (95% CI)	Controls/Cases	VE (95% CI)
Not vaccinated	149451/172793	Reference	12394/17467	Reference	4005/6954	Reference
Two doses, ≥180 days	84833/102591	-0.9% (-2-0.3)	34007/39122	18% (15.7-20.3)	15539/19463	29.6% (26.3-32.7)
Homologous booster						
Third dose of CoronaVac, 8-59 days	6058/6725	6.1% (2.6-9.5)	1238/1493	8.8% (1.1-15.9)	370/371	39.7% (29.8-48.2)
Third dose of CoronaVac, ≥60 days	2578/3567	-13.6% (-18--8.9)	7352/9141	-2.2% (-6.2-2)	11434/15139	11.8% (7.3-16.1)
Heterologous booster						
Third dose of BNT162b2, 8-59 days	108819/57507	56.4% (55.9-57)	23479/15053	56.4% (55-57.8)	4932/3273	64.2% (62-66.3)
Third dose of BNT162b2, ≥60 days	183245/156962	29.6% (28.9-30.3)	130148/116955	37.4% (35.8-38.9)	64910/64495	46.5% (44.2-48.7)
Hospitalization or Death						
Not vaccinated	1169/2894	Reference	693/2108	Reference	877/2683	Reference
Two doses, ≥180 days	732/688	67.3% (62.3-71.6)	2035/3180	49% (42.8-54.5)	2750/5207	41.2% (35.2-46.6)
Homologous booster						
Third dose of CoronaVac, 8-59 days	34/16	87% (75.2-93.2)	73/50	77.2% (65.8-84.8)	49/51	65.6% (47.5-77.5)
Third dose of CoronaVac, ≥60 days	23/21	68% (37.7-83.6)	511/462	64.1% (57.2-69.9)	2146/2964	43% (36.4-49)
Heterologous booster						
Third dose of BNT162b2, 8-59 days	775/183	91.8% (90-93.3)	1203/537	85.7% (83.4-87.7)	755/540	78.7% (75.4-81.6)
Third dose of BNT162b2, ≥60 days	1397/370	90.7% (89.1-92)	7441/3099	86.9% (85.4-88.3)	9755/7063	79.8% (77.8-81.5)

Supplementary Table 6. Characteristics of adults in Brazil, who were selected into case test negative pairs for the for the analysis of relative vaccine effectiveness analysis during the Omicron period (December 25, 2021 to Apr 22, 2022),

	**Matched pairs for Omicron period**		
	**Controls (n=620,133)**	**Cases (n=620,133)**	**SMD**
**Demographics**			
**Age, mean (SD), years**	54.23 (18.9)	54.5 (19.1)	0.016
**Age categories, n (%)**			0.058
18-39 years	185374 (29.9)	181268 (29.2)	
40-59 years	150442 (24.3)	154755 (25.0)	
60-79 years	247298 (39.9)	238998 (38.5)	
≥80 years	37019 (6.0)	45112 (7.3)	
**Male sex, n (%)**	214071 (34.5)	214071 (34.5)	<0.001
**Self-reported race[†], n (%)**			0.052
White/Branca	289223 (46.6)	293911 (47.4)	
Mixed/Pardo	178933 (28.9)	168763 (27.2)	
Black/Preta	23067 (3.7)	21092 (3.4)	
Asian/ Amarela	9970 (1.6)	9487 (1.5)	
Indigenous/Indigena	2818 (0.5)	2098 (0.3)	
Missing	116122 (18.7)	124782 (20.1)	
**Region of residence**			<0.001
North	33438 (5.4)	33438 (5.4)	
Northeast	100347 (16.2)	100347 (16.2)	
Central-West	45755 (7.4)	45755 (7.4)	
Southeast	328824 (53.0)	328824 (53.0)	
South	111769 (18.0)	111769 (18.0)	
**Reported number of chronic comorbidities[‡], n (%)**			0.014
None	546266 (88.1)	548356 (88.4)	

One or two	71314 (11.5)	68945 (11.1)	
Three or more	2553 (0.4)	2832 (0.5)	
**Prior SARS-CoV-2 exposure**			
Previous symptomatic events notified to the surveillance system¶, n (%)	164774 (26.6)	151882 (24.5)	0.124
Positive SARS-CoV-2 test result††, n (%)	40970 (6.6)	23827 (3.8)	0.127
**Interval between symptoms onset and RT-PCR testing, median (p25-p75), days**	3 [2, 4]	3 [2, 4]	0.127
**Hospitalization or Death**	15771 (2.5)	24395 (3.9)	0.079
**Vaccination status**			0.223
Two doses, ≥180 days, n (%)	120128 (19.4)	157410 (25.4)	
Third dose of CoronaVac, 0-7 days, n (%)	878 (0.1)	1140 (0.2)	
Third dose of CoronaVac, 8-59 days, n (%)	6757 (1.1)	8384 (1.4)	
Third dose of CoronaVac, 60-89 days, n (%)	5380 (0.9)	6518 (1.1)	
Third dose of CoronaVac, 90-119 days, n (%)	9410 (1.5)	12579 (2.0)	
Third dose of CoronaVac, ≥90 days, n (%	5931 (1.0)	8582 (1.4)	
Third dose of BNT162b2, 0-7 days, n (%)	14117 (2.3)	17905 (2.9)	
Third dose of BNT162b2, 8-59 days, n (%)	113848 (18.4)	74009 (11.9)	
Third dose of BNT162b2, 60-89 days, n (%)	124797 (20.1)	110356 (17.8)	
Third dose of BNT162b2, 90-119 days, n (%)	170478 (27.5)	170754 (27.5)	
Third dose of BNT162b2, ≥120 days, n (%)	48409 (7.8)	52496 (8.5)	
**Interval between second dose and RT-PCR/Antigen test, mean (SD), days**	269 [228, 305]	269 [223, 306]	0.012
**Interval between third dose and RT-PCR/Antigen test, mean (SD), days**	87 [56, 105]	91 [65, 109]	0.133

RT-PCR=reverse transcription polymerase chain reaction; SMD=standardized mean difference; SD=standard deviation; † Race/skin colour as defined by the Brazilian national census bureau (Instituto Nacional de Geografia e Estatísticas). ‡ Comorbidities included cardiovascular, or renal conditions, diabetes, chronic respiratory disorder, obesity, or immunosuppression. ¶ Reported illness with covid-19 associated symptoms in eSUS and SIVEP-Gripe databases before the start of study on 06 September 2021. †† Defined as a positive SARS-CoV-2 RT-PCR or antigen detection test result before the start of study on 06 September 2021..

Supplementary Table 7. Unadjusted vaccine effectiveness of a homologous and heterologous booster dose, relative to primary vaccination with CoronaVac during the period greater or equal to 180 days after the 2nd dose during Omicron period

	Symptomatic Covid-19		Hospitalization or Death	
	Controls/Cases	Relative VE (95% CI)	Controls/Cases	Relative VE (95% CI)
Two doses, ≥180 days	120128/157410	Reference	4360/8977	Reference
Homologous booster				
Third dose of CoronaVac, 8-59 days	6757/8384	5.3% (1.9-8.5)	148/117	59.1% (47-68.5)
Third dose of CoronaVac, 60-89 days	5380/6518	-3.1% (-6.9-0.8)	352/326	49.6% (40.2-57.5)
Third dose of CoronaVac, 90-119 days	9410/12579	-18.7% (-21.3--16.1)	964/1200	20.8% (11.7-29)
Third dose of CoronaVac, ≥120 days	5931/8582	-34.6% (-37.3--31.8)	1164/1906	-17% (-25--8)
Heterologous booster				
Third dose of BNT162b2, 8-59 days	113848/74009	53.6% (53-54.2)	2005/1243	71.5% (68.9-73.8)
Third dose of BNT162b2, 60-89 days	124797/110356	35.3% (34.6-36.1)	4804/2668	76% (74.4-77.6)
Third dose of BNT162b2, 90-119 days	170478/170754	24.3% (23.5-25.1)	7033/4653	70.3% (68.6-72)
Third dose of BNT162b2, ≥120 days	48409/52496	11.3% (9.8-12.8)	3449/3134	55.2% (51.8-58.3)

Supplementary Table 8. Characteristics of adults in Brazil, who were selected into case test negative pairs for the analysis of vaccine effectiveness during the Delta period (September 6, 2021 to December 14, 2021) and the Omicron period (December 25, 2021 to Apr 22, 2022) - **Sensitivity analysis for matching**

	**Matched pairs for Delta period**			**Matched pairs for Omicron period**		
	**Controls (n=360,045)**	**Cases (n=132,355)**	**SMD**	**Controls (n=982,144)**	**Cases (n=1,213,302)**	**SMD**
**Demographics**						
**Age, mean (SD), years**	40.4 (17.9)	44.6 (19.4)	0.225	41.7 (18.5)	42.2 (18.4)	0.025
**Age categories, n (%)**			0.227			0.034
18-39 years	222864 (61.9)	68163 (51.5)		573177 (58.4)	696053 (57.4)	
40-59 years	68145 (18.9)	27948 (21.1)		196976 (20.1)	256255 (21.1)	
60-79 years	61653 (17.1)	31947 (24.1)		187718 (19.1)	227151 (18.7))	
≥80 years	7383 (2.1)	4297 (3.2)		24273 (2.5)	33843 (2.8)	
**Male sex, n (%)**	148640 (41.3)	59822 (45.2)	0.079	388595 (39.6)	495040 (40.8)	0.025
**Self-reported race[†], n (%)**			0.071			0.087
White/Branca	165235 (45.9)	58768 (44.4)		431055 (43.9)	540505 (44.5)	
Mixed/Pardo	99280 (27.6)	37519 (28.3)		306878 (31.2)	346765 (28.6)	
Black/Preta	15917 (4.4)	5106 (3.9)		43312 (4.4)	46625 (3.8)	
Asian/ Amarela	4090 (1.1)	1861 (1.4)		17671 (1.8)	20763 (1.7)	
Indigenous/Indigena	1101 (0.3)	898 (0.7)		2800 (0.3)	2233 (0.2)	
Missing	74422 (20.7)	28203 (21.3)		180428 (18.4)	256411 (21.1)	

**Region of residence**			0.216			0.031
North	14887 (4.1)	8488 (6.4)		40809 (4.2)	55841 (4.6)	
Northeast	34766 (9.7)	15319 (11.6)		135724 (13.8)	168228 (13.9)	
Central-West	20801 (5.8)	11562 (8.7)		78255 (8.0)	89169 (7.3)	
Southeast	211159 (58.6)	64695 (48.9)		541177 (55.1)	668797 (55.1)	
South	78432 (21.8)	32291 (24.4)		186179 (19.0)	231267 (19.1)	
**Reported number of chronic comorbidities‡, n (%)**			0.122			0.014
None	325084 (90.3)	114438 (86.5)		908535 (92.5)	1126250 (92.8)	
One or two	33518 (9.3)	16900 (12.8)		71502 (7.3)	84147 (6.9)	
Three or more	1443 (0.4)	1017 (0.8)		2107(0.2)	2905 (0.2)	
**Prior SARS-CoV-2 exposure**						
Previous symptomatic events notified to the surveillance system¶, n (%)	117610 (32.7)	25033 (18.9)	0.318	307779 (31.3)	310788 (25.6)	0.127
Positive SARS-CoV-2 test result††, n (%)	24952 (6.9)	2212 (1.7)	0.261	79126 (8.1)	58724 (4.8)	0.131
**Interval between symptoms onset and RT-PCR testing, median (p25-p75), days**	3 [2, 4]	3 [2, 5]	0.143	3 [2, 4]	3 [2, 4]	0.105
**Hospitalization or death**	9980 (2.8)	14547 (11.0)	0.329	13538 (1.4)	28387 (2.3)	0.071
**Vaccination status**			0.422			0.217
Not vaccinated, n (%)	73296 (20.4)	40165 (30.3)		115379 (11.7)	168224 (13.9)	
Primary vaccination with CoronaVac						

Single dose, 0-13 days, n (%)	4477 (1.2)	2135 (1.6)		1025 (0.1)	1298 (0.1)	
Single dose, ≥14 days, n (%)	32487 (9.0)	11580 (8.7)		53933 (5.5)	64038 (5.3)	
Two doses, 0-13 days, n (%)	11889 (3.3)	3205 (2.4)		2803 (0.2)	2657(0.2)	
Two doses, 14-89 days, n (%)	92035 (25.6)	21210 (16.0)		41152 (4.2)	44794 (3.7)	
Two doses, 90-179 days, n (%)	65840 (18.3)	29431 (22.2)		268947 (27.4)	391160 (32.2)	
Two doses, ≥180 days, n (%)	47556 (13.2)	20348 (15.4)		98996 (10.1)	137720 (11.4)	
Booster vaccination						
Third dose of CoronaVac, 0-7 days, n (%)	225 (0.1)	81 (0.1)		647 (0.1)	1072 (0.1)	
Third dose of CoronaVac, 8-59 days, n (%)	1384 (0.4)	349 (0.3)		5202 (0.5)	7414 (0.6)	
Third dose of CoronaVac, ≥60 days, n (%)	241 (0.1)	61 (0.0)		12747 (1.3)	21818 (1.8)	
Third dose of BNT162b2, 0-7 days, n (%)	4041 (1.1)	1573 (1.2)		11747 (1.2)	16677 (1.4)	
Third dose of BNT162b2, 8-59 days, n (%)	24942 (6.9)	2046 (1.5)		96675 (9.8)	66339 (5.5)	
Third dose of BNT162b2, ≥60 days, n (%)	1632 (0.5)	171 (0.1)		273307 (27.8)	290091 (23.9)	
**Interval between first dose and testing, median (p25-p75), days**	34 [21, 62]	33 [19, 62]	0.032	138 [95, 169]	140 [99, 170]	0.033
**Interval between second dose and testing, median (p25-p75), days**	96 [45, 172]	145 [66, 184]	0.283	138 [115, 177]	141 [119, 176]	0.048
**Interval between third dose and testing, median (p25-p75), days**	24 [13, 41]	10 [6, 29]	0.451	86 [53, 107]	91 [64, 107]	0.073

RT-PCR=reverse transcription polymerase chain reaction; SMD=standardized mean difference; SD=standard deviation; † Race/skin colour as defined by the Brazilian national census bureau (Instituto Nacional de Geografia e Estatísticas). ‡ Comorbidities included cardiovascular, or renal conditions, diabetes, chronic respiratory disorder, obesity, or immunosuppression. . ¶ Reported illness with covid-19 associated symptoms in eSUS and SIVEP-Gripe databases before the start of study on 06 September 2021. †† Defined as a positive SARS-CoV-2 RT-PCR or antigen detection test result before the start of study on 06 September 2021

eTable 9. Sensitivity analysis of matching strategy to evaluate the adjusted vaccine effectiveness of CoronaVac and homologous or heterologous booster against symptomatic Covid-19 using RT-PCR or Antigen tests

	**Delta Period**		**Omicron Period**	
	Matching 1:1	Stratified by sets	Matching 1:1	Stratified by sets
Not vaccinated	Reference	Reference	Reference	Reference
Single dose, within 0-13 days	2.2% (-4-8.1)	4.6% (-1.3-10.2)	24.6% (19.1-29.8)	17.8% (10.1-24.9)
Single dose, ≥14 days	27.2% (25.1-29.3)	23.9% (21.8-26)	17% (15.9-18)	14.6% (13.3-15.9)
Two doses, within 0-13 days	38.5% (35.3-41.5)	35.9% (32.8-38.8)	21.3% (17.3-25.2)	27.2% (22.6-31.4)
Two doses, 14-59 days	51.3% (49.9-52.7)	48.3% (46.9-49.7)	28.1% (26.5-29.6)	26.5% (24.6-28.4)
Two doses, 60-179 days	37.6% (36.1-39.1)	33.2% (31.7-34.7)	3.9% (3.1-4.8)	0.6% (-0.4-1.6)
Two doses, ≥180 days	34% (32.3-35.7)	29.7% (27.9-31.5)	6.3% (5.3-7.3)	3.6% (2.4-4.9)
Third dose of CoronaVac, 0-7 days	44.7% (26.2-58.5)	50.4% (34.2-62.7)	9.6% (1.7-16.9)	-0.5% (-10.5-9.6)
Third dose of CoronaVac, 8-59 days	57.1% (50.4-62.9)	63.6% (58-68.5)	8.6% (5.6-11.5)	9.8% (6-13.3)
Third dose of CoronaVac, ≥60 days	53.5% (30.9-68.6)	60.1% (42.7-72.2)	-2.9% (-5.2--0.6)	-5.8% (-8.6--2.9)
Third dose of BNT162b2, 0-7 days	42.6% (38.5-46.5)	36.1% (31.6-40.3)	16.6% (14.7-18.4)	13.9% (11.5-16.1)
Third dose of BNT162b2, 8-59 days	86.7% (86-87.4)	86.4% (85.6-87.1)	56.8% (56.3-57.3)	55.2% (54.6-55.9)
Third dose of BNT162b2, ≥60 days	83.2% (79.8-86.1)	81.1% (77.2-84.3)	33.8% (33.2-34.4)	29.9% (29.1-30.7)

Supplementary Table 10. Sensitivity analysis of matching strategy to evaluate the adjusted vaccine effectiveness of CoronaVac and homologous or heterologous booster against Severe Covid-19 using RT-PCR or Antigen tests

	**Delta Period**		**Omicron Period**	
	Matching 1:1	Matching stratified sets	Matching 1:1	Matching stratified sets
Not vaccinated	Reference	Reference	Reference	Reference
Single dose, within 0-13 days	1.7% (-28.4-30.8)	22.4% (-7.8-44.5)	-3.1% (-57.5-54.7)	-64.1% (-87.3-1.7)
Single dose, ≥14 days	46.6% (38.9-53.3)	51.8% (44.9-57.8)	47.5% (40.7-53.5)	46.4% (38.9-53)
Two doses, within 0-13 days	76.7% (67.3-83.4)	79.2% (70.5-85.3)	14.8% (-46-60.8)	61.2% (22.4-80.6)
Two doses, 14-59 days	86.5% (83.4-88.9)	87.3% (84.6-89.5)	56.1% (40.6-67.5)	55.2% (38.9-67.2)
Two doses, 60-179 days	71% (68.5-73.2)	73.4% (71.1-75.5)	61.4% (57.4-65)	63.5% (59.4-67.2)
Two doses, ≥180 days	60.9% (57.3-64.2)	64.2% (60.9-67.2)	57.6% (54.4-60.6)	58.4% (54.9-61.6)
Third dose of CoronaVac, 0-7 days	80.8% (67.7-88.6)	81% (66.8-89.2)	75.6% (26.7-91.8)	79.6% (36.1-93.5)
Third dose of CoronaVac, 8-59 days	75.9% (67.8-81.9)	82.7% (77.2-86.8)	73.6% (63.9-80.7)	79.9% (72.3-85.4)
Third dose of CoronaVac, ≥60 days	75.6% (52.8-87.4)	81.7% (66.6-89.9)	67.8% (64.3-71)	72.8% (69.6-75.7)
Third dose of BNT162b2, 0-7 days	80.4% (75.8-84)	81.9% (77.6-85.4)	62.6% (50.7-71.6)	67.5% (56.5-75.7)
Third dose of BNT162b2, 8-59 days	92.3% (91-93.4)	93% (91.8-93.9)	86% (84.5-87.4)	87.1% (85.5-88.5)
Third dose of BNT162b2, ≥60 days	90.8% (84.8-94.4)	89% (82.7-93)	86.4% (85.4-87.3)	87.2% (86.1-88.1)

Supplementary Table 11. Adjusted vaccine effectiveness of CoronaVac and homologous or heterologous booster against severe Covid-19 in adults in Brazil: main analysis vs sensitivity analysis of the severe COVID-19 definition

	Delta		Omicron	
	**Hospitalization or death (Main analysis)**	**Use of Respiratory Support, ICU admission or death (Sensitivity analysis)**	**Hospitalization or death (Main analysis)**	**Use of Respiratory Support, ICU admission or death (Sensitivity analysis)**
**Not vaccinated**	Reference	Reference	Reference	Reference
**Single dose, within 0-13 days**	1.7% (-28.4-30.8)	3.9% (-33.2-38.3)	-3.1% (-57.5-54.7)	-8.5% (-69.7-63.8)
**Single dose, ≥14 days**	46.6% (38.9-53.3)	46.8% (37.8-54.5)	47.5% (40.7-53.5)	50.3% (42.1-57.4)
**Two doses, within 0-13 days**	76.7% (67.3-83.4)	82% (72-88.4)	14.8% (-46-60.8)	6.7% (-59.7-64.9)
**Two doses, 14-59 days**	86.5% (83.4-88.9)	87.5% (83.8-90.3)	56.1% (40.6-67.5)	58.7% (36.7-73.1)
**Two doses, 60-179 days**	71% (68.5-73.2)	72.6% (69.9-75.1)	61.4% (57.4-65)	63.1% (57.8-67.7)
**Two doses, ≥180 days**	60.9% (57.3-64.2)	63.7% (59.8-67.2)	57.6% (54.4-60.6)	59.1% (55.3-62.6)
**Third dose of CoronaVac, 0-7 days**	80.8% (67.7-88.6)	86.7% (75.7-92.7)	75.6% (26.7-91.8)	81.6% (21.3-95.7)
**Third dose of CoronaVac, 8-59 days**	75.9% (67.8-81.9)	78% (69.8-83.9)	73.6% (63.9-80.7)	74.3% (62.6-82.3)
**Third dose of CoronaVac, ≥60 days**	75.6% (52.8-87.4)	69.2% (34.4-85.6)	67.8% (64.3-71)	70.7% (66.8-74)
**Third dose of BNT162b2, 0-7 days**	80.4% (75.8-84)	81% (75.8-85.1)	62.6% (50.7-71.6)	68.5% (55.4-77.8)
**Third dose of BNT162b2, 8-59 days**	92.3% (91-93.4)	92.5% (91.1-93.7)	86% (84.5-87.4)	87.3% (85.6-88.8)
**Third dose of BNT162b2, ≥60 days**	90.8% (84.8-94.4)	91.1% (84-95.1)	86.4% (85.4-87.3)	87.5% (86.4-88.6)

Supplementary Table 12. Adjusted vaccine effectiveness of homologous or heterologous booster against severe Covid-19 in adults stratified by age during Omicron period in Brazil: main analysis vs sensitivity analysis of the severe COVID-19 definition

	**Hospitalization or Death (Main analysis)**			**Use of Respiratory Support, ICU admission or death (Sensitivity analysis)**		
	<60 years	60-74 years	≥75 years	<60 years	60-74 years	≥75 years
Not vaccinated	Reference	Reference	Reference	Reference	Reference	Reference
Two doses, ≥180 days	71% (65.9-75.3)	63.4% (58.3-67.9)	40.7% (33.7-46.9)	74% (67.6-79.2)	65% (59.2-70)	44.7% (37.1-51.4)
Homologous booster						
Third dose of CoronaVac, 8-59 days	86% (71.7-93.1)	80.7% (68.6-88.1)	47.7% (12.4-68.8)	91.6% (74.2-97.3)	79.7% (64.9-88.2)	53.2% (15.2-74.1)
Third dose of CoronaVac, ≥60 days	82% (60.9-91.7)	76.1% (70.8-80.4)	51.4% (44.6-57.3)	86.1% (53.5-95.9)	78.4% (72.6-83)	57.6% (50.7-63.6)
Heterologous booster						
Third dose of BNT162b2, 8-59 days	92.1% (90.2-93.7)	88.4% (86.2-90.2)	77.3% (73.1-80.8)	94% (91.7-95.7)	89.6% (87.3-91.6)	79.8% (75.4-83.4)
Third dose of BNT162b2, ≥60 days	90.2% (88.3-91.7)	90.4% (89.1-91.5)	78.5% (76.1-80.6)	93.2% (91.2-94.7)	91.2% (89.8-92.4)	81% (78.5-83.2)

Supplementary Table 13. Adjusted vaccine effectiveness of a homologous and heterologous booster dose, relative to primary vaccination with CoronaVac during the period greater or equal to 180 days after the 2nd dose during Omicron period, further adjusted by month of second dose (sensitivity analysis)

	**Symptomatic Covid-19**		**Hospitalization or Death**	
	**Main analysis**	**Sensitivity analysis adjusted by month of 2nd dose**	**Main analysis**	**Sensitivity analysis adjusted by month of 2nd dose**
	Relative VE (95% CI)	Relative VE (95% CI)	Relative VE (95% CI)	Relative VE (95% CI)
**Two doses, ≥180 days**	Reference	Reference	Reference	Reference
**Homologous booster**				
**Third dose of CoronaVac, 8-59 days**	4.9% (1.5-8.1)	1.4% (-2.1-4.8)	47.1% (27.8-61.2)	45.8% (26-60.3)
**Third dose of CoronaVac, 60-89 days**	-3.8% (-7.5-0.2)	-4.5% (-8.3--0.6)	36% (21.5-47.8)	35.8% (21.3-47.7)
**Third dose of CoronaVac, 90-119 days**	-13.8% (-16.5--11)	-14% (-16.7--11.1)	23.5% (12.4-33.1)	25.1% (14.3-34.6)
**Third dose of CoronaVac, ≥120 days**	-24.8% (-28--21.6)	-23.4% (-26.6--20.1)	20.7% (10.1-30)	24.3% (14-33.4)
**Heterologous booster**				
**Third dose of BNT162b2, 8-59 days**	53.4% (52.8-54)	51.7% (51.1-52.4)	67.3% (63.9-70.4)	66.7% (63.2-69.9)
**Third dose of BNT162b2, 60-89 days**	34.7% (34-35.5)	34.5% (33.7-35.3)	71% (68.7-73.2)	70.9% (68.5-73)
**Third dose of BNT162b2, 90-119 days**	25.2% (24.4-26)	25% (24.1-25.9)	68.6% (66.4-70.7)	69.1% (66.8-71.1)
**Third dose of BNT162b2, ≥120 days**	15.7% (14.2-17.1)	16.9% (15.4-18.5)	62.8% (59.3-65.9)	64.3% (60.9-67.5)

Supplementary Figure 3. Flow chart showing inclusion of cases and controls for the sensitivity analysis including only RT-PCR SARS-CoV-2 tests

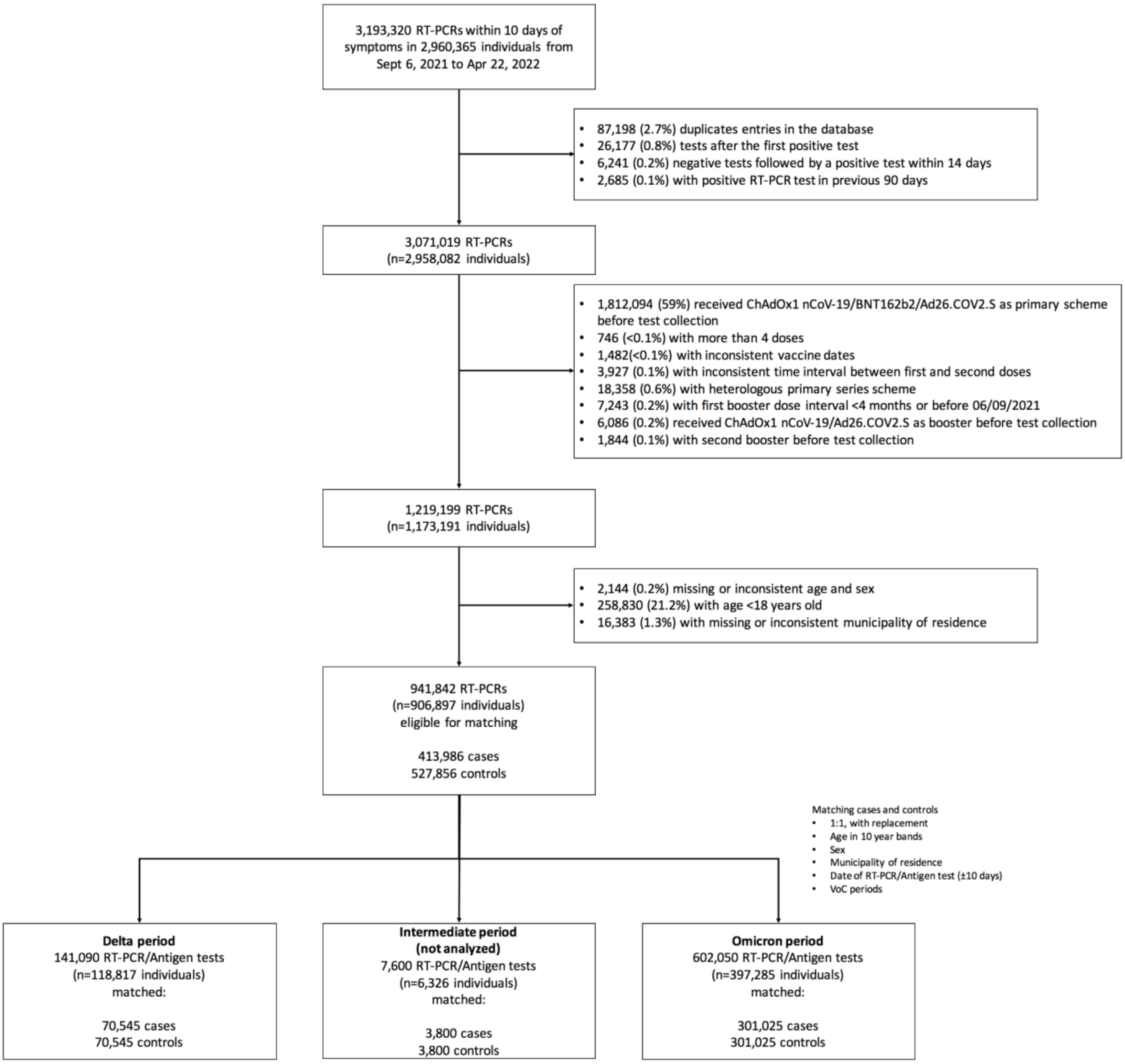

Supplementary Table 14. Characteristics of adults in Brazil, who were selected into case-test negative pairs for the sensitivity analysis including only RT-PCR tests during the Delta period (September 6, 2021 to December 14, 2021) and Omicron period (December 25, 2021 to Apr 22, 2022)

	**Matched pairs for Delta period**			**Matched pairs for Omicron period**		
	**Controls (n=70,545)**	**Cases (n=70,545)**	**SMD**	**Controls (n=301,025)**	**Cases (n=301,025)**	**SMD**
**Demographics**						
**Age, mean (SD), years**	47.4 (20.2)	47.5 (20.3)	0.007	42.7 (19)	43.0 (19.2)	0.015
**Age categories, n (%)**			0.024			0.043
18-39 years	32535 (46.1)	31990 (45.3)		173722 (57.7)	170224 (56.5)	
40-59 years	14878 (21.1)	15426 (21.9)		59687 (19.8)	63234 (21.0)	
60-79 years	19447 (27.6)	19256 (27.3)		54828 (18.2)	53014 (17.6)	
≥80 years	3685 (5.2)	3873 (5.5)		12788 (4.2)	14553 (4.8)	
**Male sex, n (%)**	32130 (45.5)	32130 (45.5)	<0.001	125368 (41.6)	125368 (41.6)	<0.001
**Self-reported race[†], n (%)**			0.092			0.094
White/Branca	28649 (40.6)	29984 (42.5)		128775 (42.8)	133966 (44.5)	
Mixed/Pardo	23789 (33.7)	21471 (30.4)		93042 (30.9)	82110 (27.3)	
Black/Preta	3498 (5.0)	2957 (4.2)		12804 (4.3)	11176 (3.7)	
Asian/ Amarela	922 (1.3)	1218 (1.7)		4694 (1.6)	5618 (1.9)	
Indigenous/Indigena	197 (0.3)	281 (0.4)		399 (0.1)	291 (0.1)	
Missing	13490 (19.1)	14634 (20.7)		61311 (20.4)	67864 (22.5)	

**Region of residence**			<0.001			<0.001
North	3424 (4.9)	3424 (4.9)		8355 (2.8)	8355 (2.8)	
Northeast	10216 (14.5)	10216 (14.5)		50186 (16.7)	50186 (16.7)	
Central-West	5758 (8.2)	5758 (8.2)		15313 (5.1)	15313 (5.1)	
Southeast	38529 (54.6)	38529 (54.6)		199212 (66.2)	199212 (66.2)	
South	12618 (17.9)	12618 (17.9)		27959 (9.3)	27959 (9.3)	
**Reported number of chronic comorbidities[‡], n (%)**			0.037			0.064
None	58550 (83.0)	57585 (81.6)		265890 (88.3)	271794 (90.3)	
One or two	11258 (16.0)	12088 (17.1)		32894 (10.9)	27565 (9.2)	
Three or more	737 (1.0)	872 (1.2)		2241 (0.7)	1666 (0.6)	
**Prior SARS-CoV-2 exposure**						
Previous symptomatic events notified to the surveillance system[¶], n (%)	17825 (25.3)	12137 (17.2)	0.198	88840 (29.5)	78548 (26.1)	0.076
Positive SARS-CoV-2 test result[††], n (%)	3881 (5.5)	1402 (2.0)	0.186	24261 (8.1)	16613 (5.5)	0.101
**Interval between symptoms onset and RT-PCR testing, median (p25-p75), days**	3 [2, 5]	3 [2, 5]	0.115	3 [2, 4]	3 [2, 4]	0.151
**Hospitalization or Death**	8097 (11.5)	12842 (18.2)	0.191	26664 (8.9)	19851 (6.6)	0.085
**Vaccination status**			0.294			0.223
Not vaccinated, n (%)	14100 (20.0)	19354 (27.4)		31413 (10.4)	36865 (12.2)	
Single dose, within 0-13 days, n (%)	676 (1.0)	956 (1.4)		301 (0.1)	305 (0.1)	

Single dose, ≥14 days, n (%)	5695 (8.1)	5810 (8.2)		14809 (4.9)	14667 (4.9)	
Two doses, within 0-13 days, n (%)	1904 (2.7)	1697 (2.4)		796 (0.3)	601 (0.2)	
Two doses, 14-89 days, n (%)	13248 (18.8)	10877 (15.4)		10758 (3.6)	9808 (3.3)	
Two doses, 90-179 days, n (%)	17605 (25.0)	17009 (24.1)		84500 (28.1)	100216 (33.3)	
Two doses, ≥180 days, n (%)	11431 (16.2)	12351 (17.5)		30159 (10.0)	35974 (12.0)	
Third dose of CoronaVac, 0-7 days, n (%)	68 (0.1)	54(0.1)		411 (0.1)	368 (0.1)	
Third dose of CoronaVac, 8-59 days, n (%)	328 (0.5)	271(0.4)		2416 (0.8)	2545 (0.8)	
Third dose of CoronaVac, ≥60 days, n (%)	54(0.1)	47(0.1)		6412 (2.1)	8309 (2.8)	
Third dose of BNT162b2, 0-7 days, n (%)	929 (1.3)	837(1.2)		3913 (1.3)	4132 (1.4)	
Third dose of BNT162b2, 8-59 days, n (%)	42421(6.0)	1187 (17)		29257 (9.7)	17102 (5.7)	
Third dose of BNT162b2, ≥60 days, n (%)	265 (0.4)	95(0.1)		85880 (28.5)	70133 (23.3)	
**Interval between first dose and testing, median (p25-p75), days**	36 [23, 67]	35 [21, 67]	0.006	138 [93, 170]	143 [101, 173]	0.072
**Interval between second dose and testing, median (p25-p75), days**	144 [60, 181]	150 [73, 187]	0.105	140 [119, 176]	142 [122, 178]	0.039
**Interval between third dose and testing, median (p25-p75), days**	21 [11, 38]	10 [6, 30]	0.307	86 [53, 103]	90 [62, 106]	0.123

RT-PCR=reverse transcription polymerase chain reaction; SMD=standardized mean difference; SD=standard deviation; † Race/skin colour as defined by the Brazilian national census bureau (Instituto Nacional de Geografia e Estatísticas). ‡ Comorbidities included cardiovascular, or renal conditions, diabetes, chronic respiratory disorder, obesity, or immunosuppression. ¶ Reported illness with covid-19 associated symptoms in eSUS and SIVEP-Gripe databases before the start of study on 06 September 2021. †† Defined as a positive SARS-CoV-2 RT-PCR or antigen detection test result before the start of study on 06 September 2021.

Supplementary Table 15. Adjusted vaccine effectiveness of CoronaVac and homologous or heterologous booster against symptomatic Covid-19 and Covid-19 hospital admission or deaths in adults in Brazil, from the sensitivity analysis including RT-PCR SARS-CoV-2 tests only

	**Symptomatic COVID-19**		**Hospitalization or death**	
	Delta	Omicron	Delta	Omicron
**Not vaccinated**	Reference	Reference	Reference	Reference
**Single dose, within 0-13 days**	-11.9% (-20.8--2)	7.8% (-7.8-21.6)	-16.5% (-49.1-27.1)	-
**Single dose, ≥14 days**	25.5% (22-28.8)	10.5% (7.9-12.9)	45.2% (36-53.1)	47.1% (38.7-54.3)
**Two doses, within 0-13 days**	34.5% (29.3-39.2)	30.7% (22.7-37.8)	78.2% (67-85.6)	67.8% (15.3-87.8)
**Two doses, 14-59 days**	49% (46.8-51.2)	22.2% (18.2-26)	85.4% (81.4-88.5)	23% (-15.7-50)
**Two doses, 60-179 days**	35.5% (33.1-37.8)	-7.1% (-8.9--5.3)	68.1% (65-71)	53.4% (47.2-58.9)
**Two doses, ≥180 days**	30.1% (27.3-32.8)	7% (4.8-9.1)	57.3% (52.7-61.5)	55.8% (51.8-59.5)
**Third dose of CoronaVac, 0-7 days**	52.3% (31-67.1)	18.2% (5.4-29.2)	76% (57.6-86.4)	18.4% (-72.3-81.6)
**Third dose of CoronaVac, 8-59 days**	56.9% (47.8-64.4)	10.5% (5-15.7)	74.1% (64.5-81.1)	72.1% (59.7-80.7)
**Third dose of CoronaVac, ≥60 days**	64.1% (41.4-78)	-1.6% (-5.8-2.8)	84.2% (67.5-92.3)	63.6% (58.9-67.7)
**Third dose of BNT162b2, 0-7 days**	46.4% (40.4-51.7)	6.9% (2.3-11.2)	78.5% (72.5-83.2)	63.8% (48.8-74.4)
**Third dose of BNT162b2, 8-59 days**	85% (83.8-86.2)	53.5% (52.3-54.7)	90.5% (88.7-92)	84.4% (82.3-86.2)
**Third dose of BNT162b2, ≥60 days**	84.8% (79.9-88.5)	39.6% (38.4-40.8)	87.3% (78.1-92.6)	82.9% (81.4-84.3)

Supplementary Table 16. Characteristics of adults in Brazil, who were selected into case-test negative pairs for the sensitivity analysis using RT-PCR SARS-CoV-2 tests only, during the Omicron period (December 25, 2021 to Apr 10, 2022), for the analysis of relative vaccine effectiveness

	**Matched pairs for Omicron period**		
	**Controls (n=135,053)**	**Cases (n=135,053)**	**SMD**
**Demographics**			
**Age, mean (SD), years**	54.1 (19.4)	54.2 (19.7)	0.006
**Age categories, n (%)**			0.042
18-39 years	41810 (31.0)	41514 (30.7)	
40-59 years	34262 (25.4)	34931 (25.9)	
60-79 years	48057 (35.6)	46309 (34.3)	
≥80 years	10924 (8.1)	12299 (9.1)	
**Male sex, n (%)**	46477 (34.4)	46477 (34.4)	<0.001
**Self-reported race[†], n (%)**			0.126
White/Branca	61832 (45.8)	63243 (46.8)	
Mixed/Pardo	38848 (28.8)	32776 (24.3)	
Black/Preta	5334 (3.9)	4561 (3.4)	
Asian/ Amarela	2067 (1.5)	2350 (1.7)	
Indigenous/Indigena	194 (0.1)	176 (0.1)	
Missing	26778 (19.8)	31947 (23.7)	
**Region of residence**			<0.001
North	3859 (2.9)	3859 (2.9)	
Northeast	24618 (18.2)	24618 (18.2)	
Central-West	7255 (5.4)	7255 (5.4)	
Southeast	86825 (64.3)	86825 (64.3)	
South	12496 (9.3)	12496 (9.3)	
**Reported number of chronic comorbidities[‡], n (%)**			0.087

None	110250 (81.6)	114600 (84.9)	
One or two	22967 (17.0)	19083 (14.1)	
Three or more	1836 (1.4)	1370 (1.0)	
**Prior SARS-CoV-2 exposure****			
Previous symptomatic events notified to the surveillance system¶, n (%)	41841 (31.0)	34182 (25.3)	0.126
Positive SARS-CoV-2 test result††, n (%)	10419 (7.7)	6303 (4.7)	0.127
**Interval between symptoms onset and RT-PCR testing, median (p25-p75), days**	3 [2, 4]	3 [1, 4]	0.164
**Hospitalization or Death**	18040 (13.4)	13484 (10.0)	0.106
**Vaccination status**			0.218
Two doses, ≥180 days, n (%)	26610 (19.7)	34817 (25.8)	
Third dose of CoronaVac, 0-7 days, n (%)	262 (0.2)	357 (0.3)	
Third dose of CoronaVac, 8-59 days, n (%)	1929 (1.4)	2458 (1.8)	
Third dose of CoronaVac, 60-89 days, n (%)	1613 (1.2)	1858 (1.4)	
Third dose of CoronaVac, 90-119 days, n (%)	2694 (2.0)	3811 (2.8)	
Third dose of CoronaVac, ≥120 days, n (%)	1888 (1.4)	2561 (1.9)	
Third dose of BNT162b2, 0-7 days, n (%)	2649 (2.0)	3954 (2.9)	
Third dose of BNT162b2, 8-59 days, n (%)	23637 (17.5)	16503 (12.2)	
Third dose of BNT162b2, 60-89 days, n (%)	26921 (19.9)	23643 (17.5)	
Third dose of BNT162b2, 90-119 days, n (%)	38587 (28.6)	36377 (26.9)	
Third dose of BNT162b2, ≥120 days, n (%)	8263 (6.1)	8714 (6.5)	
**Interval between second dose and RT-PCR/Antigen test, mean (SD), days**	272 [239, 309]	273 [237, 313]	0.003
**Interval between third dose and RT-PCR/Antigen test, mean (SD), days**	88 [57, 104]	90 [63, 106]	0.078

RT-PCR=reverse transcription polymerase chain reaction; SMD=standardized mean difference; SD=standard deviation; † Race/skin colour as defined by the Brazilian national census bureau (Instituto Nacional de Geografia e Estatísticas). ‡ Comorbidities included cardiovascular, or renal conditions, diabetes, chronic respiratory disorder, obesity, or immunosuppression. ** Before the start of study on 06 September 2021. ¶ Reported illness with covid-19 associated symptoms in eSUS and SIVEP-Gripe databases. †† Defined as a positive SARS-CoV-2 RT-PCR or antigen detection test result

Supplementary Table 17. Adjusted vaccine effectiveness of homologous or heterologous booster against symptomatic Covid-19 and Covid-19 hospital admission or deaths in adults stratified by age during Omicron period in Brazil from the sensitivity analysis including RT-PCR SARS-CoV-2 tests only

	**Symptomatic COVID-19**			**Hospitalization or Death**		
	<60 years	60-74 years	≥75 years	<60 years	60-74 years	≥75 years
Not vaccinated	Reference	Reference	Reference	Reference	Reference	Reference
Two doses, ≥180 days	-3.6% (-6.2--1)	34.1% (30-38)	23.6% (17.3-29.4)	72% (65.4-77.4)	63.8% (57.8-69)	31.5% (22.2-39.7)
Homologous booster						
Third dose of CoronaVac, 8-59 days	9.2% (2.9-15)	24.4% (11.1-35.6)	21.8% (-2.2-40.2)	91.4% (78-96.6)	74.3% (55.3-85.2)	51.1% (8.8-73.8)
Third dose of CoronaVac, ≥60 days	-15.7% (-23.9--6.7)	23% (16.3-29.2)	3.6% (-4.8-11.6)	78.4% (44.1-91.6)	73.1% (65.7-78.9)	40.9% (31.5-49)
Heterologous booster						
Third dose of BNT162b2, 8-59 days	51.5% (50.1-52.8)	63.3% (60.6-65.8)	66.6% (62.5-70.2)	91.9% (89.2-93.9)	86.8% (83.7-89.3)	74.2% (68.6-78.8)
Third dose of BNT162b2, ≥60 days	40% (38.6-41.3)	48.6% (45.7-51.4)	42.8% (38.4-46.8)	93.9% (92.3-95.2)	87.6% (85.6-89.4)	70.2% (66.4-73.6)

Supplementary Table 18. Adjusted Vaccine effectiveness of homologous and heterologous booster relative to those at least 180 days after the second dose of a primary series of CoronaVac during the Omicron period, from the sensitivity analysis including RT-PCR SARS-CoV-2 tests only

	**Symptomatic COVID-19**	**Hospitalization or Death**
	**Relative VE (95% CI)**	**Relative VE (95% CI)**
Two doses, ≥180 days	Reference	Reference
**Homologous booster**		
Third dose of CoronaVac, 8-59 days	1.5% (-5.0-7.7)	37% (7.5-57.1)
Third dose of CoronaVac, 60-79 days	2.7% (-4.7-9.7)	34.6% (17.2-48.4)
Third dose of CoronaVac, 90-119 days	-19.3% (-24--14.4)	7.8% (-7.6-21.5)
Third dose of CoronaVac, ≥120 days	-20.6% (-26.4--14.3)	15.9% (3.2-27)
**Heterologous booster**		
Third dose of BNT162b2, 8-59 days	49.8% (48.5-51.2)	62.8% (57.9-67.1)
Third dose of BNT162b2, 60-89 days	35.9% (34.3-37.5)	68.3% (65.1-71.1)
Third dose of BNT162b2, 90-119 days	30.9% (29.2-32.4)	62.0% (58.8-64.9)
Third dose of BNT162b2, ≥120 days	16.6% (13.2-19.9)	50.3% (44.8-55.3)
