# Supplementary material for: Effectiveness of an inactivated Covid-19 vaccine with homologous and heterologous boosters against Omicron in Brazil
Source: Nat Commun. 2022 Oct 6;13:5536. doi: 10.1038/s41467-022-33169-0 (PMC9537178; doi:10.1038/s41467-022-33169-0)
Supplement: Supplementary file 2 — Reporting Summary [file 41467_2022_33169_MOESM2_ESM.pdf]

nature research

Corresponding author(s): Otavio Ranzani

Last updated by author(s): 2022/08/08

Reporting Summary

Nature Research wishes to improve the reproducibility of the work that we publish. This form provides structure for consistency and transparency in reporting. For further information on Nature Research policies, see our Editorial Policies and the Editorial Policy Checklist.

Statistics

For all statistical analyses, confirm that the following items are present in the figure legend, table legend, main text, or Methods section.

n/a	Confirmed	
☐	☒	The exact sample size (n) for each experimental group/condition, given as a discrete number and unit of measurement
☐	☒	A statement on whether measurements were taken from distinct samples or whether the same sample was measured repeatedly
☒	☐	The statistical test(s) used AND whether they are one- or two-sided<br>*Only common tests should be described solely by name; describe more complex techniques in the Methods section.*
☐	☒	A description of all covariates tested
☒	☐	A description of any assumptions or corrections, such as tests of normality and adjustment for multiple comparisons
☐	☒	A full description of the statistical parameters including central tendency (e.g. means) or other basic estimates (e.g. regression coefficient) AND variation (e.g. standard deviation) or associated estimates of uncertainty (e.g. confidence intervals)
☒	☐	For null hypothesis testing, the test statistic (e.g. F, t, r) with confidence intervals, effect sizes, degrees of freedom and P value noted<br>*Give P values as exact values whenever suitable.*
☒	☐	For Bayesian analysis, information on the choice of priors and Markov chain Monte Carlo settings
☒	☐	For hierarchical and complex designs, identification of the appropriate level for tests and full reporting of outcomes
☒	☐	Estimates of effect sizes (e.g. Cohen's d, Pearson's r), indicating how they were calculated

Our web collection on statistics for biologists contains articles on many of the points above.

Software and code

Policy information about availability of computer code

Data collection	Data were collected routinely by the Ministry of Health.
Data analysis	We used R version 4.1.2 to perform all data analyses. Code used to conduct the statistical analysis is available in the repository https://github.com/juliocroda/VebraCOVID-19

For manuscripts utilizing custom algorithms or software that are central to the research but not yet described in published literature, software must be made available to editors and reviewers. We strongly encourage code deposition in a community repository (e.g. GitHub). See the Nature Research guidelines for submitting code & software for further information.

Data

Policy information about availability of data

All manuscripts must include a data availability statement. This statement should provide the following information, where applicable:

- Accession codes, unique identifiers, or web links for publicly available datasets
- A list of figures that have associated raw data
- A description of any restrictions on data availability

Deidentified databases as well as the R codes will be deposited in the repository https://github.com/juliocroda/VebraCOVID-19. For Fig. 1, vaccine data was obtained from OpenDataSUS (https://opendatasus.saude.gov.br/, access date 2022-05-09) and variant data from GISAID (https://www.gisaid.org/hcov19-variants/, access date 2022-05-09).

Field-specific reporting

Please select the one below that is the best fit for your research. If you are not sure, read the appropriate sections before making your selection.

☒ Life sciences ☐ Behavioural & social sciences ☐ Ecological, evolutionary & environmental sciences

For a reference copy of the document with all sections, see nature.com/documents/nr-reporting-summary-flat.pdf

Life sciences study design

All studies must disclose on these points even when the disclosure is negative.

Sample size	We did not perform a formal power analysis for this study. Based on our previous studies, we expected to have enough power because of the large sample size due to the Omicron outbreak in Brazil.
Data exclusions	We describe exclusion criteria in the methods, and detail numbers excluded in the flowchart in eFigure 2. We excluded individuals with missing or inconsistent information on age, sex, municipality of residence, and on vaccination and testing status and dates. We excluded RT-PCR/antigen tests that were not collected within 10 days of symptom onset to avoid potentially misclassification, positive or negative RT-PCR/ antigen tests with a positive RT-PCR/antigen test in the previous 90 days to capture only incident infections and avoid a second positive test because of prolonged viral shedding, and negative RT-PCR/antigen tests with a positive RT-PCR/antigen test occurring in the following 14 days because of likely false-negative test in the first negative test. For individuals who received multiple RT-PCR or antigen tests during the study period, we included all eligible tests up to and including the first positive test.
Replication	We performed four sensitivity analyses with different matching schemes, RT-PCR tests which produced similar findings to the primary analysis.
Randomization	This is an observational study, so no randomization occurred. We used a matched test-negative study to reduce bias, and adjusted in the study analysis using conditional logistic regression with stratification.
Blinding	This was a retrospective study, so blinding was not possible.

Reporting for specific materials, systems and methods

We require information from authors about some types of materials, experimental systems and methods used in many studies. Here, indicate whether each material, system or method listed is relevant to your study. If you are not sure if a list item applies to your research, read the appropriate section before selecting a response.

Materials & experimental systems

n/a	Involved in the study	
☒	☐	Antibodies
☒	☐	Eukaryotic cell lines
☒	☐	Palaeontology and archaeology
☒	☐	Animals and other organisms
☐	☒	Human research participants
☒	☐	Clinical data
☒	☐	Dual use research of concern

Methods

n/a	Involved in the study	
☒	☐	ChIP-seq
☒	☐	Flow cytometry
☒	☐	MRI-based neuroimaging

Human research participants

Policy information about studies involving human research participants

Population characteristics	The average age of participants was 43 years, with 42% being male. 44% of individuals self-reported white race, and 8% had at least one reported comorbidity.
Recruitment	The study population was adults (aged ≥18 years) residing in Brazil, and who underwent SARS-CoV-2 RT-PCR or rapid antigen testing associated with symptomatic illness14 during the study period. We excluded individuals with missing or inconsistent information on age, sex, municipality of residence, and on vaccination and testing status and dates. We excluded RT-PCR/ antigen tests that were not collected within 10 days of symptom onset to avoid potentially misclassification, positive or negative RT-PCR/antigen tests with a positive RT-PCR/antigen test in the previous 90 days to capture only incident infections and avoid a second positive test because of prolonged viral shedding, and negative RT-PCR/antigen tests with a positive RT-PCR/antigen test occurring in the following 14 days because of likely false-negative test in the first negative test. For individuals who received multiple RT-PCR or antigen tests during the study period, we included all eligible tests up to and including the first positive test (ie, the first positive test in the study period and at least 90 days prior to another positive).
Ethics oversight	This study was approved by the ethical committee for research of Federal University of Mato Grosso do Sul (CAAE: 43289221.5.0000.0021)

Note that full information on the approval of the study protocol must also be provided in the manuscript.
